# Acute adrenal insufficiency due to paracoccidiodomycosis. Report of 2 cases

**DOI:** 10.1016/j.mmcr.2020.08.001

**Published:** 2020-08-13

**Authors:** Juan Camilo Motta, Edgar Camilo Barrera

**Affiliations:** a Internal Medicine Resident, Universidad del Rosario, Bogotá, Colombia; b Department of Internal Medicine, Fundación Cardioinfantil, Bogota, Colombia

**Keywords:** Adrenal insufficiency, Brain tumour, Immunocompetent, Disseminated paracoccidiodomycosis

## Abstract

Paracoccidiodomycosis s is an endemic infection in Latin America. It can affect several organs, but systemic involvement is rare, especially when the adrenal glands and the central nervous system are affected. We describe two cases of paracoccidiodomycosis presenting with Addison’s disease, one of which also presented with a pseudotumor cerebri. The diagnosis of paracoccidiodomycosis was confirmed in both cases with histopathological studies. Antifungal management and hormone supplementation were given, achieving complete resolution of symptoms. 2012 Elsevier Ltd. All rights reserved.

## 1 Introduction

Paracoccidiodomycosis is a systemic mycosis, which is caused by a *Paracoccidioides complex*, which includes five species that are endemic from Mexico (south of North America), Central America and South America [1,2]. Nearly 80% of cases of this infection are reported in Brazil, followed by Venezuela, Colombia, Argentina and Ecuador [[3], [4], [5]]. In Colombia, its incidence ranges from 0.35 to 3.08 per 100,000 persons [3,6].

Chronic paracoccidiodomycosis occurs in 74% to 96% of cases. It is more frequent in men aged 30 to 60 years, and the lung is the most affected organ (90% of cases), followed by the upper airways [1,4]. It can affect the adrenal glands in up to 44% patients [1,7]. Acute adrenal crisis has been reported only in 2.9% of cases in Colombia and 14% in Brazil [8,9]; the central nervous system (CNS) manifestation is also rare, causing meningitis, myelitis or brain tumour [7,10].

In this paper, we present the cases of two patients with adrenal insufficiency due to paracoccidiodomycosis, one of whom also developed a pseudotumor cerebri as an unusual manifestation of this infection.

## 2 Case presentations

### 2.1 Case 1

A 69-year-old male from Las Piedras, Tolima, Colombia, presented with a 3-month history of weight loss (20 kg), asthenia and abdominal pain with a feeling of early fullness, at the emergency department (day 0). The patient worked in the construction industry and had a history of heavy smoking (60 cigarette packs/year). Physical examination upon admission, heart rate: 73 bpm; blood pressure: 100/50 mmHg; no other abnormal findings were observed. Laboratory investigations showed renal failure, mild hyponatremia and hyperkalaemia (sodium 130 meq/L, potassium 5.5 meq/L, creatinine 1.6 mg/dl) normal CBC and liver enzymes. Differential diagnosis upon admission was lung/gastric cancer versus AIDS.

**Fig. 1:**
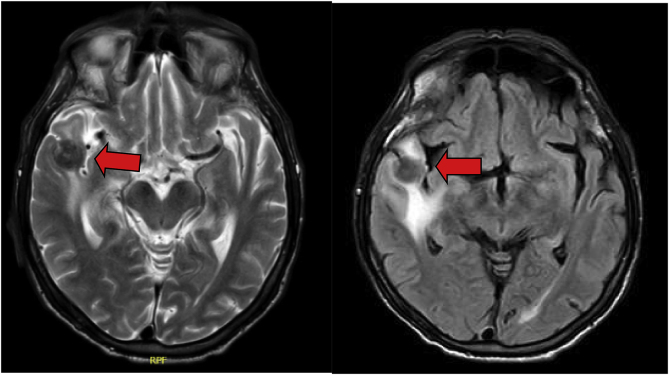
**(Case 1) Day +2** Brain MRI (T2 and FLAIR). Multiple focal cortical and subcortical lesions diffusely distributed in the supratentorial and infratentorial regions, and the left pontine (red arrows). (For interpretation of the references to colour in this figure legend, the reader is referred to the Web version of this article.)

**Fig. 2:**
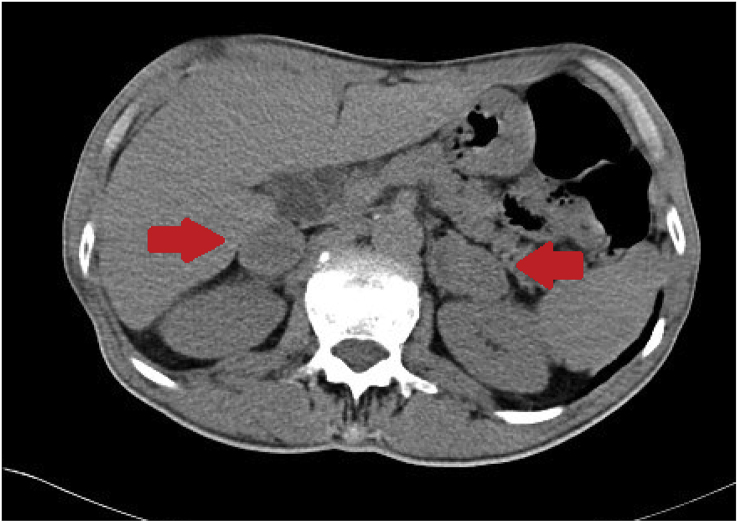
**(Case 1) Day +3** Abdominal CT. Bilateral enlargement of adrenal glands bilaterally with some central hypodense areas (red arrows). (For interpretation of the references to colour in this figure legend, the reader is referred to the Web version of this article.)

During observation, he had several generalized tonic clonic seizures, so a brain MRI was performed, showing several cortical and subcortical lesions (Fig. 1). A neoplasm diagnosis was suspected, and extended imaging studies with chest and abdomen CT scans were performed. They revealed an adrenal mass greater than 4 cm with a heterogeneous density (Fig. 2). Guided biopsies were taken from the adrenal glands and brain day +8. HIV and VDRL were negative.

The patient developed refractory hypotension required vasopressor management with noradrenaline. He was transferred to the intensive care unit, where a broad-spectrum antibiotic management was started, considering the possibilities of septic shock, worsening kidney acute renal failure, hyponatremia and hyperkalaemia (sodium 124 meq/L, potassium 6.3 meq/L, creatinine 1.9 mg/dl), which did not improve despite treatment on day + 10.

Primary adrenal insufficiency was suspected, and a blood cortisol test was performed. It was 11.8 mcg/dl, in the grey zone, and further studies were requested with the following results: Dehydroepiandrosterone sulphate (DHEA-Sulphate) levels: 4.28 μg/ml (33.6–248); adrenocorticotropic hormone (ACTH) levels: 69.5 pg/ml (7.2–63.3); androstenedione levels: 0.33 ng/ml (0.5–3.5). Prednisolone and fludrocortisone were started, and low blood pressure episodes and hydro electrolytic disorders resolved, confirming the diagnosis.

**Fig. 3:**
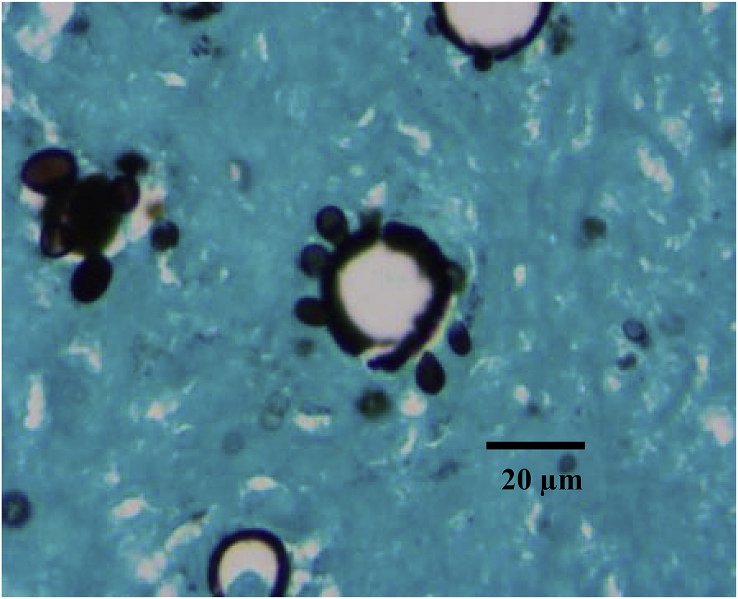
**(Case 1) Day +18** Adrenal tissue biopsy in which the Grocott-Gomori's methenamine silver staining method was used. Mariner’s wheel like round yeasts with multiple blastoconidia of narrow base are observed, which allowed reaching the *paracoccidioidomycosis* diagnosis.

**Fig. 4:**
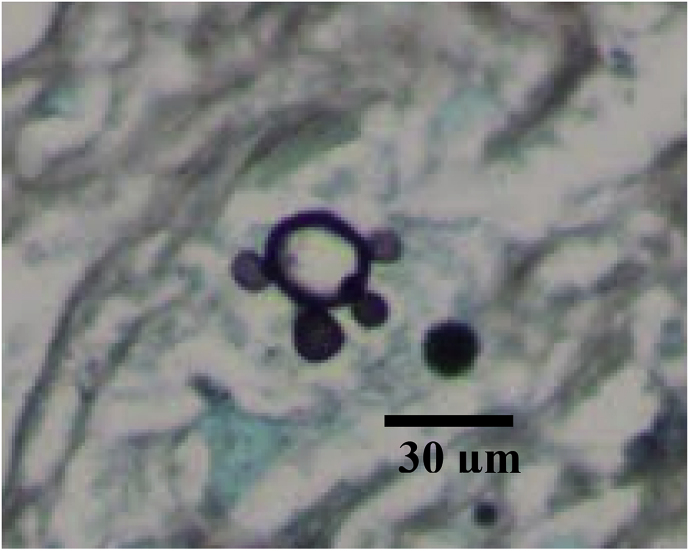
**(Case 1) Day +18** Brain biopsy image showing a mariner’s wheel appearance fungal structure compatible with *Paracoccidioides complex.*

On day +18, adrenal and brain biopsies showed the mariners’ wheel sign, suggesting fungal structures of *Paracoccidioides spp*. Infection was confirmed through the Grocott-Gomori's methenamine silver stain method (Fig. 3 and Fig. 4).

Directed treatment was administered with amphotericin B, doses 3 mg/kg/day (144 mg) for 14 days as induction therapy, followed by itraconazole 200 mg daily for 6 months, prednisolone 7.5 mg daily, and fludrocortisone 0.05 mg once daily. Upon treatment completion, the patient’s clinical signs and symptoms completely disappeared; the patient was discharged and asymptomatic on day +32.

### 2.2 Case 2

A 69-year-old male, farmer was admitted to the emergency department (day 0) due to a six-month involuntary weight loss (15 kg), asthenia, fatigue, diffuse abdominal pain, recurrent vomiting episodes and nausea-the latter of which had persisted for the past week prior to consultation. He was born in Miranda, Boyacá, Colombia, but lives in Sopó, Cundinamarca, Colombia. The patient had a history of heavy smoking (20 cigarette packs/year). An outpatient CT scan, performed one month prior to consultation, showed an adrenal mass. On admission to the emergency department, differential diagnosis was neoplasia and less likely adrenal tuberculosis.

Physical examination on admission: heart rate: 108 bpm; blood pressure: 80/50 mmHg; hyperpigmentation; no other abnormal findings were observed. Intravenous fluid management was started with persisted hypotension. Laboratory investigations showed renal failure, hyponatremia and hyperkalaemia (sodium 125 meq/L, potassium 6.7 meq/L, creatinine 4.1 mg/dl). Anaemia, thrombocytopenia and differential diagnoses were ruled out.

On day +5, acute adrenal insufficiency diagnosis was suspected, and several tests were taken. Cortisol levels of 5.1 μg/dl (3.7–19.4) and adrenocorticotropic hormone (ACTH) levels of 97.6 pg/ml (7.2–63.3) confirmed the diagnosis. Intravenous hydrocortisone 50 mg every 6 h was started with resolution of hypotension and electrolyte disorders.

**Fig. 5:**
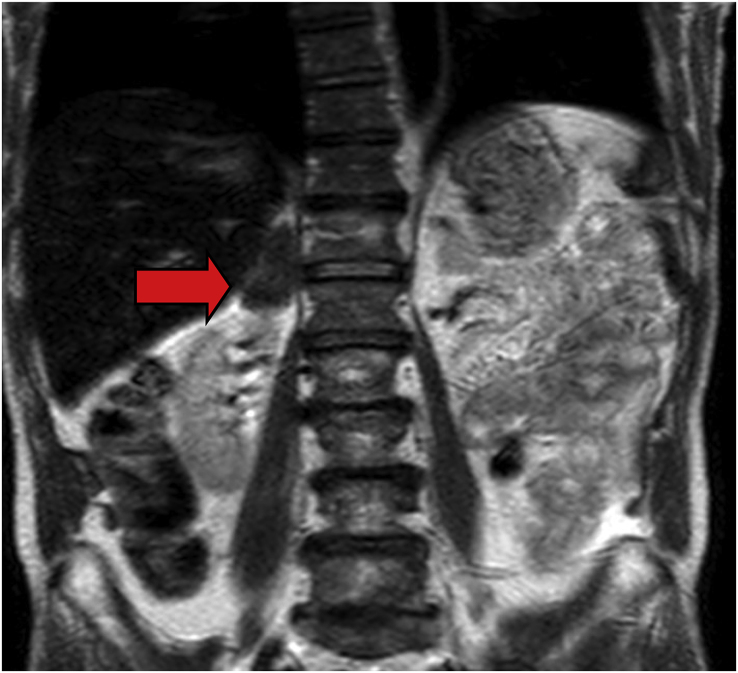
**(Case 2) Day +6** Abdominal MRI: 33 mm diameter right adrenal focal lesion, which shows intermediate signal intensity at T2 and T1. The lesion is not associated with intra- or extra-cellular "fat" and presents restricted diffusion. (Red Arrow). (For interpretation of the references to colour in this figure legend, the reader is referred to the Web version of this article.)

**Fig. 6:**
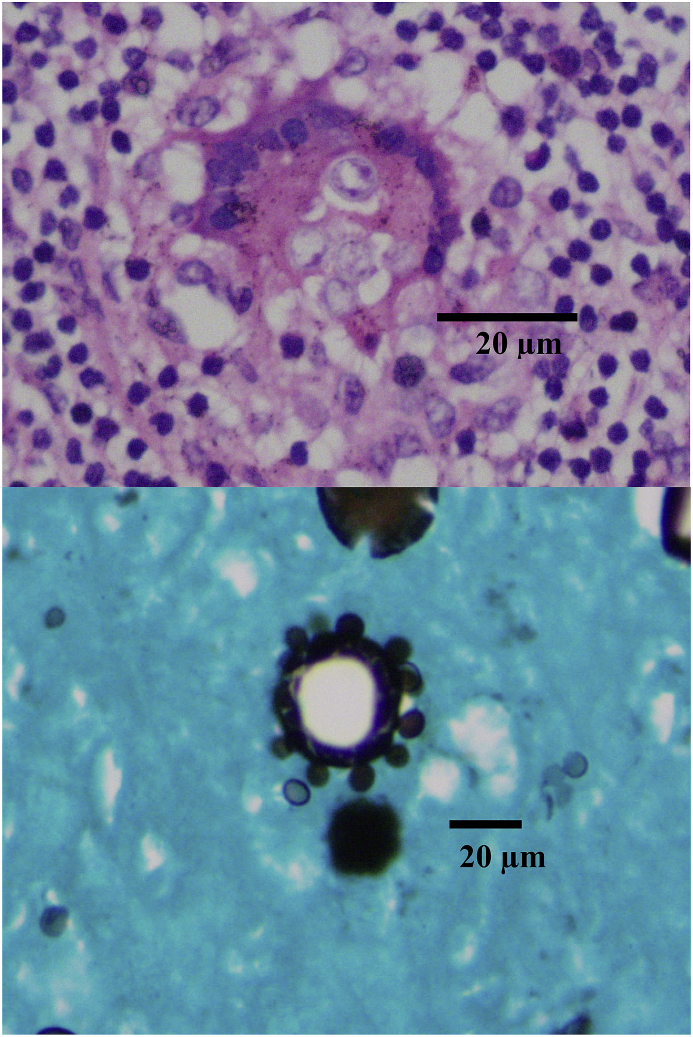
**(Case 2) Day +16** Granulomas H&E stain and mariner’s wheel like fungal structures of different sizes (paracoccidiodomycosis), Grocott-Gomori's methenamine silver stain.

To clarify the aetiology and ambulatory CT findings, an abdominal MRI was performed. The adrenal mass had radiological features that suggested a tumour (Fig. 5). Surgical resection and biopsy were indicated on day +8. The histopathological report was a granuloma with mariner’s wheel sign, compatible with paracoccidiodomycosis infection, which was confirmed through the Grocott-Gomori's methenamine silver stain method (Fig. 6) on day + 16.

With diagnosis confirmation, a 14-day treatment was started with prednisolone 10 mg once daily and fludrocortisone 0.1 mg once daily. It was considered a moderate disease, so itraconazole 200 mg once daily was started. Patient showed a clinical response on day +30 and was discharged. A 6-month itraconazole 200 mg once daily treatment was indicated. In outpatient follow-up visits, the patient was asymptomatic.

## 3 Discussion

We describe two cases of paracoccidiodomycosis presenting with adrenal insufficiency due to paracoccidiodomycosis, a rare manifestation. Paracoccidiodomycosis is the only endemic mycosis which had been associated with affecting the adrenal glands. Multiple histopathological samples were obtained, which confirmed the diagnosis in both cases, but the absence of molecular tests limited the clinical approach.

Paracoccidiodomycosis was first described in 1908 in Brazil by Adolfo Lutz, when he treated two patients with skin lesions [11,12]. Then, in 1912, Splendore found that the pathogen was a dimorphic fungus, and in 1930 [12,13]. Almeida described the taxonomy and morphology of the fungus, naming it *Paracoccidioides brasiliensis* [[11], [12], [13]].

Paracoccidiodomycosis mostly affects men aged 30 to 60 years old, with a 15:1 ratio compared to women [7,13,14]. This could be explained because men usually work in agriculture, gardening and construction, activities which are carried out where the fungus contaminates the soil [1,4,15].

According to the International Paracoccidiodomycosis Colloquium, held in Medellín, Colombia, in 1986, this disease has three forms: the acute and subacute types, which mostly affect children and adolescents; and the chronic type, which occurs in adults and affects the mucous membranes and the lungs as a reactivation of the primary and residual infections [1,16].

Chronic paracoccidiodomycosis is the most common type. It has been reported in 74% to 96% of cases [1,4] and may affect only a single organ. The upper airways and lung involvement are described in 80% of these cases [1,4,5]. Systemic paracoccidiodomycosis cases are rare and are most frequently associated with immunosuppression by any cause [4,14].

This fungal infection rarely spreads to the adrenal glands and causes Addison's disease [[7], [8], [9]]. In Colombia, Oñate et al. [8] published a case series in 2002 describing 207 cases of paracoccidiodomycosis. Only six patients (2.9%) presented with adrenal insufficiency, and all of them were male farmers and smokers, aged 48 to 75 years old. Similar characteristics to those of the patients in this study were presented; however, in the series, all patients had lung involvement [8].

CNS involvement ranges from 3.4% to 25.4% [10,13]. In Colombia, there are few reported paracoccidiodomycosis cases with CNS involvement. In 1973, Saravia et al. published the largest case series with six cases with post-mortem diagnosis and five patients with pseudotumor cerebri [17]. Therefore, more common differential diagnoses, such as brain metastases, toxoplasmosis, and cysticercosis, should always be considered first [1,10].

The treatment consists of daily oral administration of itraconazole 200 mg for 12 months when mild or moderate disease is diagnosed [1,4]. For systematic and severe involvement, the use of amphotericin B during 2 to 4 weeks is preferred as an induction therapy. Doses of 0.5–0.7 mg/kg/day for conventional amphotericin B (Deoxycholate) and 3–5 mg/kg/day amphotericin B liposomal injections are indicated [1,4,15]. Prednisolone and fludrocortisone supplementation must be given for primary adrenal insufficiency [18].

In conclusion, paracoccidiodomycosis is endemic in Latin America and is a diagnosis that should be considered when male patients are presenting with adrenal insufficiency, especially when an adrenal mass is detected.

## Declaration of competing interest

No Conflict of Interest.
